# Natural variation in the transcriptional response of *Drosophila melanogaster* to oxidative stress

**DOI:** 10.1093/g3journal/jkab366

**Published:** 2021-10-25

**Authors:** Timothy J S Ramnarine, Sonja Grath, John Parsch

**Affiliations:** Division of Evolutionary Biology, Faculty of Biology, Ludwig-Maximilians-Universität (LMU) München, Planegg-Martinsried 82152, Germany

**Keywords:** adaptation, gene expression, metallothionein, population genetics, transcriptomics

## Abstract

Broadly distributed species must cope with diverse and changing environmental conditions, including various forms of stress. Cosmopolitan populations of *Drosophila melanogaster* are more tolerant to oxidative stress than those from the species’ ancestral range in sub-Saharan Africa, and the degree of tolerance is associated with an insertion/deletion polymorphism in the 3′ untranslated region of the *Metallothionein A* (*MtnA*) gene that varies clinally in frequency. We examined oxidative stress tolerance and the transcriptional response to oxidative stress in cosmopolitan and sub-Saharan African populations of *D. melanogaster*, including paired samples with allelic differences at the *MtnA* locus. We found that the effect of the *MtnA* polymorphism on oxidative stress tolerance was dependent on the genomic background, with the deletion allele increasing tolerance only in a northern, temperate population. Genes that were differentially expressed under oxidative stress included *MtnA* and other metallothioneins, as well as those involved in glutathione metabolism and other genes known to be part of the oxidative stress response or the general stress response. A gene coexpression analysis revealed further genes and pathways that respond to oxidative stress including those involved in additional metabolic processes, autophagy, and apoptosis. There was a significant overlap among the genes induced by oxidative and cold stress, which suggests a shared response pathway to these two stresses. Interestingly, the *MtnA* deletion was associated with consistent changes in the expression of many genes across all genomic backgrounds, regardless of the expression level of the *MtnA* gene itself. We hypothesize that this is an indirect effect driven by the loss of microRNA binding sites within the *MtnA* 3′ untranslated region.

## Introduction 

Adaptation of a species to changes in environmental conditions caused by seasonal variation, climate change, or range expansion is necessary for survival and proliferation. Understanding the process of such adaptation is an intrinsic goal of evolutionary biologists, however, a major challenge is the identification of adaptive traits and their underlying molecular and genetic bases. *Drosophila melanogaster*, due to its experimental versatility, well-detailed genetics, and broad geographic distribution, has become a leading model organism for studying the molecular basis of adaptation and environment-based selection. Previous work on this topic typically has involved genomic comparisons between *D. melanogaster* populations from the ancestral (sub-Saharan Africa) and derived (cosmopolitan) species ranges (Harr *et al.* 2002; Ometto *et al.* 2005; Hutter *et al.* 2008; Glaser-Schmitt and Parsch 2018; Voigt *et al.* 2019), and may include an environmental gradient such as a latitudinal cline (Sezgin *et al.* 2004; Bergland *et al.* 2016; Juneja *et al.* 2016; Cogni *et al.* 2017; Erickson *et al.* 2020).

One example of a clinally varying genetic polymorphism is a 49-bp insertion/deletion (indel) polymorphism in the 3′ untranslated region (UTR) of the *Metallothionein A* (*MtnA*) gene (Catalán *et al.* 2016). The deletion represents the derived allele: it is rare in sub-Saharan African populations and absent in closely related *Drosophila* species (Lange *et al.* 1990; Theodore *et al.* 1991; Stephan *et al.* 1994). In non-African populations, the deletion is common and increases in frequency with distance from the equator in Europe, North America, and Australia (Catalán *et al.* 2016; Ramnarine *et al.* 2019). Functionally, the deletion is associated with increased *MtnA* expression and greater oxidative stress tolerance (Catalán *et al.* 2016; Ramnarine *et al.* 2019). These observations suggest that the *MtnA* deletion is selectively favored, at least in temperate regions, due to a beneficial effect of providing protection against oxidative stress. Another cosmopolitan polymorphism, *Bari-Juvenile hormone epoxy hydrolase* (*Bari-Jheh*), which is a transposable element insertion associated with changes in the regulation of the *Juvenile hormone epoxide hydrolase* (*Jheh*) genes and increased oxidative stress tolerance (González *et al.* 2009; Guio *et al.* 2014), is also present at high frequency in temperate populations. In *Drosophila* species, temperate populations have been shown to be more cold tolerant than ancestral, tropical populations (Svetec *et al.* 2011; von Heckel *et al.* 2016; Königer and Grath 2018). Given that both the *MtnA* deletion and *Bari-Jheh* insertion are more common in regions that experience cold temperatures and large seasonal fluctuations in temperature, it is possible that thermal variability contributes directly to oxidative stress (Joanisse and Storey 1996, 1998; Harrison *et al.* 2001; Klok *et al.* 2004; Lalouette *et al.* 2011; MacMillan *et al.* 2012), and/or that these two forms of stress induce a common response pathway.

In this study, we examine oxidative stress tolerance and the transcriptional response to oxidative stress in inbred lines derived from cosmopolitan (Germany, Cyprus, and Malaysia) and sub-Saharan African (Zambia) populations of *D. melanogaster*. For the cosmopolitan populations, we compare paired samples that differ in the presence of the *MtnA* 3′ UTR deletion, including nearly isogenic lines from Germany and Cyprus in which the remainder of the genome has been homogenized. We find that the *MtnA* deletion is associated with increased oxidative stress tolerance in the German background, but not the Cypriot or Malaysian background, which highlights the importance of genetic background on the physiological effects of the *MtnA* deletion. We detected a strong transcriptional response to oxidative stress in all lines, including the up-regulation of *MtnA* expression, which is consistent with it playing a protective role against oxidative stress. There was significant overlap between the genes up-regulated during oxidative stress and those up-regulated by cold stress, although there were also many genes that responded specifically to one type of stress or the other. Across all backgrounds the presence of the *MtnA* deletion led to a greater transcriptional response to oxidative stress, which may be caused by the loss of microRNA (miRNA) binding sites within the deleted region of the 3′ UTR, increasing the availability of the miRNAs to regulate other genes.

## Materials and methods

### Fly culturing and establishment of nearly isogenic lines

Isofemale lines from Munich, Germany (M9 and M12) and Nicosia, Cyprus (C2) were established from wild-caught flies collected in 2014 (Kapun *et al.* 2020). Each line was derived from a single female and maintained by mass sib–sib mating at each generation (in vials with approximately 50–100 flies transferred per generation). After the initial establishment of the isofemale lines, the *MtnA* 3′ UTR deletion was found to segregate within these lines, as determined by PCR-genotyping of individual flies (Catalán *et al.* 2016). These lines were maintained in the same way for an additional 50–60 generations. Subsequently, individual males and females within each isofemale line were mated to generate new inbred lines homozygous for either the deletion or the nondeletion allele. The deletion status of each new inbred line was verified by genotyping both parents, as well as a pool of 10 of their offspring. We additionally examined two isofemale lines from Kuala Lumpur, Malaysia, which had previously been shown to differ in their *MtnA 3*′ UTR allele (KL1, deletion; KL2, nondeletion) (Catalán *et al.* 2016), and four isofemale lines from the ancestral range of the species in Siavonga, Zambia (ZI197, ZI254, ZI273, and ZI418) (Pool *et al.* 2012). All of the Zambian lines had the ancestral, nondeletion *MtnA* allele (Catalán *et al.* 2016; Ramnarine *et al.* 2019). In the following, lines with the *MtnA* deletion are indicated with a “Δ” at the end of their line name. All stocks were maintained on cornmeal-yeast-molasses medium at 21°C and a 14 h light/10 h dark cycle.

### Oxidative stress tolerance assays

Previously, using *Drosophila* genetic reference panel (DGRP) lines (Mackay *et al.* 2012) we detected a significant association between the *MtnA* deletion and increased tolerance to menadione sodium bisulfite (MSB)-induced stress (Ramnarine *et al.* 2019). Therefore, to determine the effects of genetic background and the *MtnA* 3′ UTR deletion on oxidative stress tolerance, we assayed the survivorship of adult flies of each line after exposure to MSB (Sigma-Aldrich, Munich, Germany). For each line, males (age 4–6 days) were separated from culture and sorted into replicate groups of 20 flies per vial on standard food (cornmeal-yeast-molasses medium) for 1 day to acclimate. On the following day, 3–5 replicates from each line were transferred to “control” vials containing standard fly food and 5–16 replicates from each line were transferred to “stress” vials containing 75 mM MSB-dosed cornmeal-yeast-molasses medium. Assays were performed in tolerance chambers at 25°C with a 14 h light/10 h dark cycle. Mortality was recorded as the proportion of dead flies in the stress treatment compared to the control after 48 h.

### RNA extraction and sequencing

To measure the transcriptional response to oxidative stress, we repeated the MSB stress tolerance assays described above. The German, Malaysian, and Cypriot lines were exposed to stress (or control) condition for 24 h and then total RNA was extracted from two biological replicates per line. Each replicate used 60 whole heads from living male flies. The Zambian flies under both treatments were exposed for a shorter time (18–20 h) to compensate for their high mortality and total RNA was extracted from heads in two replicates per line as described above. This design resulted in a total of 48 samples (12 lines × 2 treatments × 2 biological replicates) that were used for transcriptome profiling. Total RNA was extracted using the RNeasy Mini Kit (Qiagen, Hilden, Germany) following the manufacturer’s protocol. Poly-A mRNA purification, reverse transcription, and high-throughput sequencing were performed by Novogene (Cambridge, UK) using a Novaseq 6000 sequencer (Illumina, San Diego, CA, USA) to generate 150-bp paired-end reads. From the RNA-sequencing we obtained between 29.3 and 67.6 million reads per library, and an average of 97% of these reads were successfully mapped to the *D. melanogaster* transcriptome (FlyBase, release 6.31) (Thurmond *et al.* 2019) using NextGenMap (version 0.5.5) (Sedlazeck *et al.* 2013). The resulting alignments were used to estimate pairwise sequence divergence between strains, considering only sites with a minimum coverage of 20x in both strains.

### Detection of differentially expressed genes

Differentially expressed genes were called with DESeq2 (version 1.28.1) (Love *et al.* 2014) as implemented in R (version 4.0.0) (R Core Team 2020). Significant differences between control and stress samples were determined using a Wald test and the *P*-values subsequently corrected for multiple testing according to Benjamini and Hochberg (1995). Genes with an adjusted *P*-value less than 0.05 were considered to be differentially expressed. Initially we performed unsupervised clustering (DESeq2 design: “∼population + genotype + treatment”) on the variance-stabilized data and visualized the results with a principal component analysis (PCA). The PCA revealed an extreme outlier, which was one of the Munich (M9) nondeletion stress replicates (Supplementary Figure S1). This sample was excluded from further analyses. To identify genes that shared a common change in expression after MSB exposure across all genetic backgrounds, we focused on the treatment factor and applied a design using only “∼treatment.” Lists of overall up- and downregulated differentially expressed genes associated to treatment (Supplementary Tables S1 and S2) were then generated for subsequent gene ontology (GO) analysis. These lists were also used in comparisons with two external transcriptomic datasets involving cold stress (von Heckel *et al.* 2016) and oxidative stress (Weber *et al.* 2012). For this, the log_2_ fold-change values of the genes matching the list of genes associated to oxidative stress (Supplementary Figures S2 and S3) and cold tolerance (Supplementary Figures S4 and S5) were compared. The significance of the overlap between the oxidative stress response and the cold response genes was determined by a hypergeometric test.

### Gene ontology analysis

GO analysis was performed using FlyMine (Lyne *et al.* 2007) to determine the number of significantly up-regulated and downregulated genes matching a GO term, and REVIGO (Supek *et al.* 2011) to merge redundant GO terms. A Holm–Bonferroni correction was applied to calculate adjusted *P*-values (Supplementary Tables S3 and S4). Information from FlyBase (release FB2019_06) (Thurmond *et al.* 2019) and AMIGO (Carbon *et al.* 2009) was used to cross-reference genes matching significant GO terms with their annotated function.

### Weighted gene coexpression network analysis

To construct gene networks and identify highly relevant (hub) genes and pathways involved in the response to oxidative stress, we applied weighted gene coexpression analysis (WGCNA) (Zhang and Horvath 2005) as implemented in the R package “WGCNA” (version 1.69) using R (version 3.6.3) (Langfelder and Horvath 2008). The WGCNA approach allows for the detection of expression modules or pathways in which many genes show small but consistent changes and, therefore, complements the analysis of individual genes (GO analysis) by means of gene enrichment analysis. In the WGCNA workflow, normalized expression data are used to cluster genes that show similar expression across samples, indicating that the expression of the clustered genes is correlated. Such genes are referred to as being topologically correlated. Coexpression modules are defined by hierarchical clustering of these topological clusters modules. By convention, each module is assigned a color and genes that do not show any coexpression with other genes are placed in the “gray” module. After module identification, the module expression (eigengene) is used to associate the module with categorical information. We performed WGCNA with two design formulas for differential gene expression analysis: (1) considering only the effect of treatment (∼treatment) to build on the identified genes and pathways identified in the GO analysis, and (2) incorporating the effect of the *MtnA* indel (∼genotype + treatment + genotype: treatment) to identify differentially expressed genes in response to oxidative stress associated to the deletion background relative to the nondeletion background.

### Comparison of *MtnA* deletion and nondeletion lines

To determine how the indel polymorphism in the *MtnA* 3′ UTR influenced gene expression and the response to oxidative stress, we applied the DESeq2 “∼treatment” design (described above) within each cosmopolitan background separately and then performed the following pairwise comparisons: deletion stress *vs* deletion control, nondeletion stress *vs* nondeletion control, deletion stress *vs* nondeletion stress and deletion control *vs* nondeletion control (Supplementary Table S6). Adjusted *P*-values were determined as described above, and genes with an adjusted *P < *0.05 were considered as differentially expressed between deletion and nondeletion lines within each treatment. This approach was particularly suited to the comparison of the nearly isogenic lines (Germany and Cyprus) and paired population samples (Malaysia), as it allowed for the comparison of *MtnA* genotypes without the extensive background variation present in between-strain or between-population comparisons.

### Expression analysis of potential miRNA target genes

To determine if some of the expression differences observed between *MtnA* deletion and nondeletion lines could be caused by the differential binding of miRNAs, we examined the expression of conserved target genes associated with miRNAs predicted to bind within the 49-bp region that is deleted in *MtnA* 3′ UTR (Catalán *et al.* 2016). The target genes of these miRNAs were identified using TargetScanFly (version 7.2) (Agarwal *et al.* 2018). Because miRNAs are typically involved in the negative regulation of gene expression, we focused on the predicted target genes that were downregulated under oxidative stress. We then examined the expression of these genes using a direct contrast in DESeq2 (version 1.28.1) (Love *et al.* 2014) between deletion and nondeletion lines in the cosmopolitan backgrounds in response to stress (the Zambian lines were excluded because they were fixed for the nondeletion allele). A paired sample Wilcoxon test was applied to test for a directional disparity in the expression of these genes between deletion and nondeletion lines. Finally, the resulting list of genes was tested for functional enrichments using FlyMine (Lyne *et al.* 2007).

## Results

### Variation in oxidative stress tolerance among lines and populations

**Figure 1:**
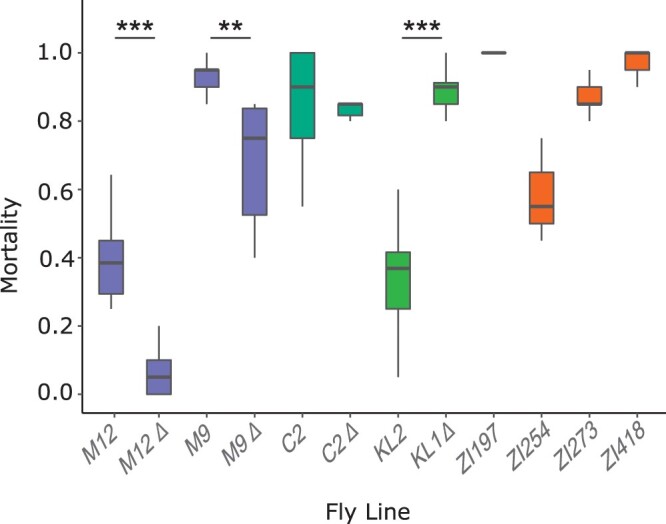
Mortality after 48 h of exposure to MSB-dosed food in lines originating from: Munich, Germany (M9 and M12); Nicosia, Cyprus (C2); Kuala Lumpur, Malaysia (KL), and Siavonga, Zambia (ZI). Differences between *MtnA* genotypes within the same background were tested with a Wilcoxon test. **P < 0.01, ***P < 0.001.

To assay oxidative stress tolerance of the different genotypes and populations, adult males (4–6 days post-eclosion) were placed on food containing 75 mM MSB and their mortality recorded over 48 h. As a control, males of the same age were placed on standard food. Since we observed no mortality on the standard food, we assume that any mortality observed on the MSB food is a consequence of MSB-induced oxidative stress and not any other aspect of the rearing conditions. A comparison of nearly isogenic lines differing specifically at the *MtnA* locus found that the 3′ UTR deletion was associated with increased oxidative stress tolerance in the German background, but not in the Cypriot background (Figure  1). When comparing the two isofemale lines from Malaysia, the *MtnA* deletion was associated with a reduction in oxidative stress tolerance. Thus, the effect of the *MtnA* 3′ UTR deletion on oxidative stress tolerance appears to be highly dependent on the genomic background and is variable among cosmopolitan populations. The Zambian lines, all of which had the ancestral (nondeletion) *MtnA* allele, were relatively susceptible to oxidative stress. However, there were differences in mortality among the Zambian lines, which suggests that genetic variation for oxidative stress tolerance is also segregating within the ancestral range of the species.

### The transcriptional response to oxidative stress

**Figure 2:**
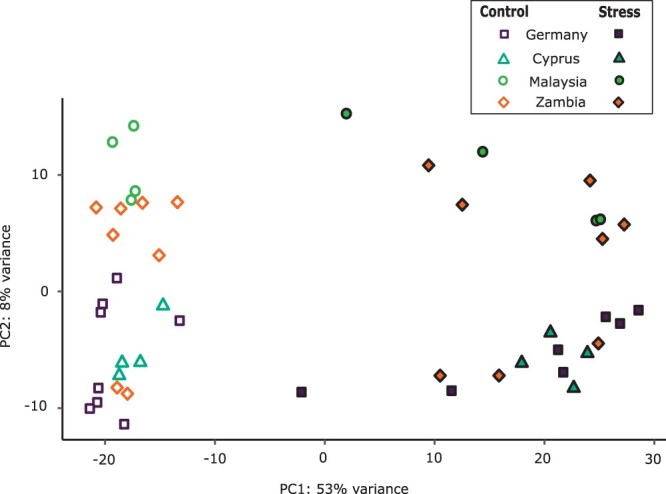
PCA plot showing the amount of variation explained by treatment and population of origin: Munich, Germany (M9 and M12); Nicosia, Cyprus (C2); Kuala Lumpur, Malaysia (KL), and Siavonga, Zambia (ZI). Filled symbols represent the MSB-stress samples, while open symbols represent the control samples.

To investigate the transcriptional response to oxidative stress, we performed high-throughput RNA sequencing (RNA-seq) on the heads of male flies exposed to MSB-dosed and standard food. Across all samples, we found a strong transcriptional response to MSB treatment, with 45% of all expressed genes being differentially expressed in response to oxidative stress (false discovery rate = 5%). In total, 3434 (25%) genes were up-regulated after stress treatment (Supplementary Table S1), while 2839 (20%) were downregulated (Supplementary Table S2). A PCA showed that the majority of the variance (53%) could be explained by the treatment (PC1) (Figure  2), while the second principal component (PC2) mainly separates the samples by population and accounts for only 8% of the variance.

### Functional enrichment of oxidative stress response genes

A GO enrichment analysis of the genes showing a general up- or downregulation of expression in stressed samples relative to the controls revealed various processes broadly related to metabolism and stress response (Supplementary Tables S3 and S4), with the strongest enrichments (ranked by *P*-value) being for categories related to metabolic processes (Table  1). In addition, we observed a large number of genes in the categories “proteolysis” (up-regulated; *P *=* *2.51E-05) and “animal organ development” (downregulated; *P *=* *3.23E-11).

**Table 1:** Top five GO enrichment terms for genes up- or downregulated under oxidative stress

Regulation	GO term	Description	*P*-value	Genes
Up	0051186	Cofactor metabolic process	1.52E−08	98
Up	0006749	Glutathione metabolic process	3.08E−08	36
Up	0006950	Response to stress	2.81E−06	393
Up	0006575	Cellular modified amino acid metabolic process	4.78E−06	48
Up	0006508	Proteolysis	2.51E−05	301
Down	0044281	Small molecule metabolic process	3.12E−13	264
Down	0046034	ATP metabolic process	2.75E−11	77
Down	0048513	Animal organ development	3.23E−11	368
Down	0032543	Mitochondrial translation	1.78E−08	56
Down	0006091	Generation of precursor metabolites and energy	1.18E−07	91

A Holm–Bonferroni correction was applied to the *P*-values.

### Expression of candidate genes associated with oxidative stress tolerance

In a previous quantitative trait locus study, seven genes (*fog*, *nACRα-30D*, *ena*, *rg*, *CG9650*, *Eip75B*, and *hbn*) that were significantly associated with oxidative stress susceptibility were functionally validated by comparing wild-type and mutant alleles (Weber *et al.* 2012). Of these seven genes, only one was significantly differentially expressed in our dataset (*Eip75B*), and it shows a relatively small difference in expression between treatments (log_2_ fold-change* *=* *0.27; Supplementary Figure S2). Thus, there is little evidence that these genes play a role in the oxidative stress response of our samples, at least at the transcriptional level. We further examined the expression of genes associated to epoxide hydrolase, specifically the *Jheh* genes. The *Bari-Jheh* transposon, which is present at high frequencies in temperate regions, has been shown to have a *cis*-regulatory effect on the expression of nearby genes, leading to up-regulation of *Jheh1* and *Jheh2* and downregulation of *Jheh3* under oxidative stress conditions (Guio *et al.* 2014). In our dataset, we observe the same relationship among these three genes in response to MSB (Supplementary Figure S3), which further supports their functional role in mediating oxidative stress tolerance.

### Comparison of genes induced by oxidative stress and cold stress

Previous studies suggested that there may be a common response to both cold and oxidative stress (Lalouette *et al.* 2011; MacMillan *et al.* 2016). To investigate this possibility, we compared the genes responding to oxidative stress in our study to those responding to cold stress in a previous study that compared the transcriptional response of European and African strains of *D. melanogaster* (von Heckel *et al.* 2016). For this, we focused on a time point of 90 min post-recovery after a 7-h cold shock, which shows the strongest response in terms of gene expression (Colinet *et al.* 2010; von Heckel *et al.* 2016). At this time point, 1535 genes were up-regulated and 1979 genes were downregulated in response to cold shock (von Heckel *et al.* 2016). Of these genes, a significant proportion overlaps with the differentially expressed genes induced by MSB as determined by a hypergeometric test, including 881 up-regulated (*P *=* *1.4E-187) and 832 shared downregulated (*P *=* *1.7E-126) genes. The study of von Heckel *et al.* (2016) identified 16 genes that respond to cold shock in a population-specific way (Europe *vs* Africa), indicating a genotype-by-environment interaction. In our data, 11 of these 16 genes showed a significant change in expression under oxidative stress (Supplementary Figure S4). Furthermore, of the 47 cold tolerance candidate genes identified by von Heckel *et al.* (2016), 34 are significantly differentially expressed under oxidative stress in our dataset (Supplementary Figure S5).

### Coexpressed gene modules in response to oxidative stress

**Figure 3:**
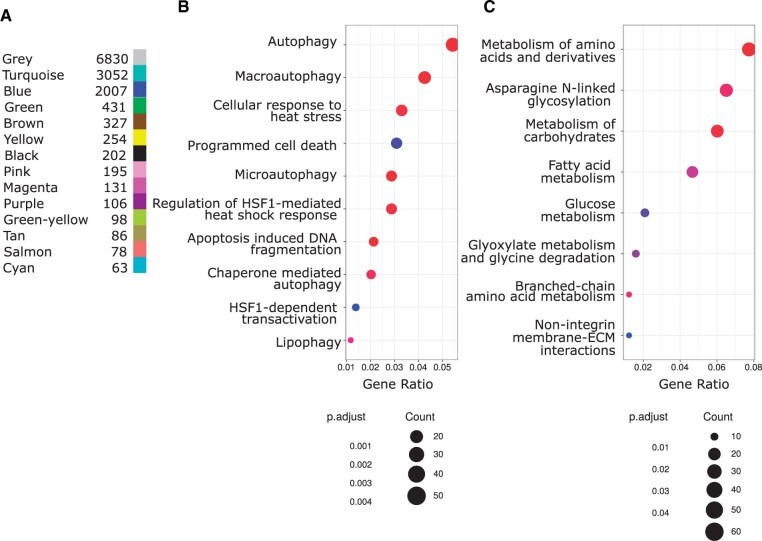
Weighted gene coexpression network analysis (WGCNA): (A) Number of genes in the final merged modules, (B) Reactome dotplot of enriched terms for the turquoise module, (C) Reactome dotplot of enriched terms for the blue module. *P*-values (“p.adjust”) are adjusted according to the method of Benjamini and Hochberg (1995). The number of genes in a pathway is encoded in the dot size (count).

Modules of coexpressed genes were identified with weighted gene coexpression network analysis (WGCNA) as implemented in the R package WGCNA by Langfelder and Horvath (2008). WGCNA called 13 modules that contained 7030 genes in total, while an additional 6830 genes were gathered in the gray module, which contains genes that have not been clustered in any module (*i.e.*, unassigned genes; Figure  3). Using an ANOVA, we determined that five modules (turquoise, blue, green, pink, and magenta) out of the original 13 modules showed significant association with treatment (*P < *0.001). Two of these modules (turquoise and blue) contained the majority of the associated genes. The turquoise module (3052 genes) corresponds to up-regulation of autophagy categories as well as heat-shock response and programmed cell death, which are components of the generic extra- and/or intra-cellular stress response (Figure  3). Expectedly, there is a strong interconnection of the categories in this module to several heat shock protein (HSP) genes (Supplementary Figure S6A). The blue module (2007 genes) is associated with several metabolic processes that are downregulated in response to stress (Figure  3). Two genes within this module exhibit a particularly large negative fold-change: *CG16700* and *MFS9* (*Major Facilitator Superfamily Transporter 9*), both of which are involved in transmembrane activity (Gaudet *et al.* 2011). An additional three genes within this module also stand out because they are connected to multiple sub-modules, and therefore are likely to affect more pathways: *Got1* (*Glutamate oxaloacetate transaminase 1*), *aralar1* (*CG2139*; a member of the mitochondrial carrier family of genes), and *CG18003* (*Hao*). *Got1* and *aralar1* are functionally involved in glutamate activity (Gaudet *et al.* 2011) and connect the categories involving metabolism of carbohydrates, glucose, and amino acids. *CG18003* (*Hao*), which is predicted to have (S)-2-hydroxy-acid oxidase activity, connects fatty acid metabolism and amino acid metabolism (Supplementary Figure S6B).

### The effect of the *MtnA* indel polymorphism on gene expression

**Figure 4:**
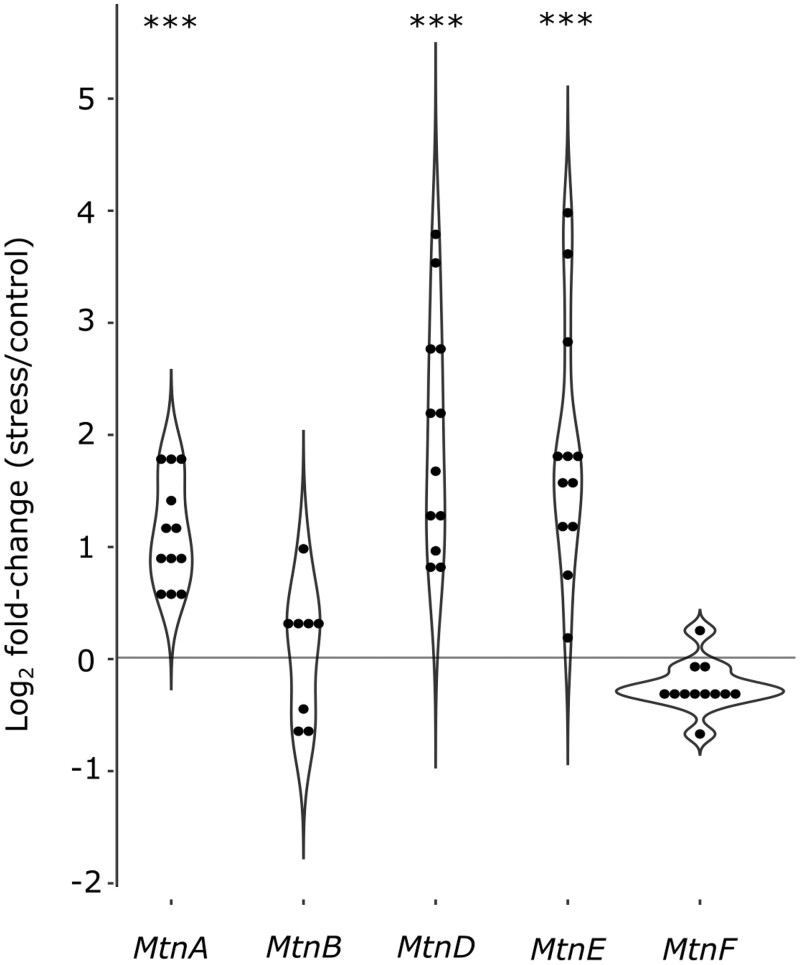
MSB-induced log_2_ fold-change in expression (stress/control) of the metallothionein group genes *MtnA*, *MtnB*, *MtnD*, *MtnE*, and *MtnF*. The points within each violin plot represent the values of the different inbred lines. Values for *MtnC* and some data points for *MtnB* are not included, as expression was below the detection threshold. Asterisks indicate the adjusted *P*-value from a Wald test as applied in DESeq2 in stress *vs* control comparisons across all lines. ***P < 0.001.

**Figure 5:**
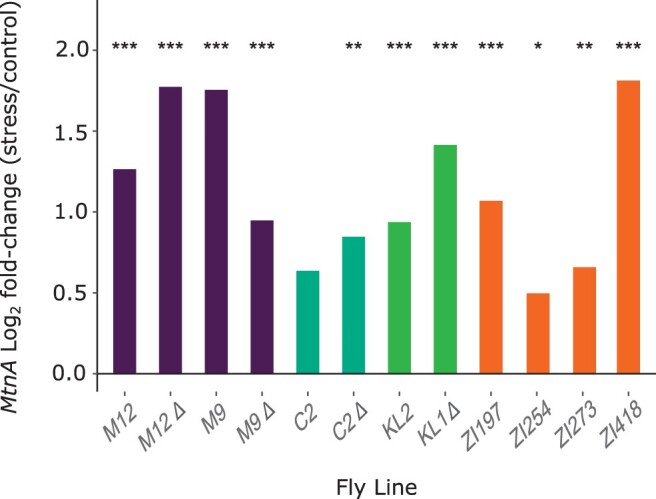
Log_2_ fold-change in *MtnA* expression induced by oxidative stress in different backgrounds: Munich, Germany (M9 and M12); Nicosia, Cyprus (C2); Kuala Lumpur, Malaysia (KL), and Siavonga, Zambia (ZI). Lines homozygous for the *MtnA* 3′ UTR deletion are denoted by “Δ.” Asterisks indicate the adjusted *P*-value from a Wald test as applied in DESeq2. * *P < *0.05, **P < 0.01, ***P < 0.001.

**Figure 6:**
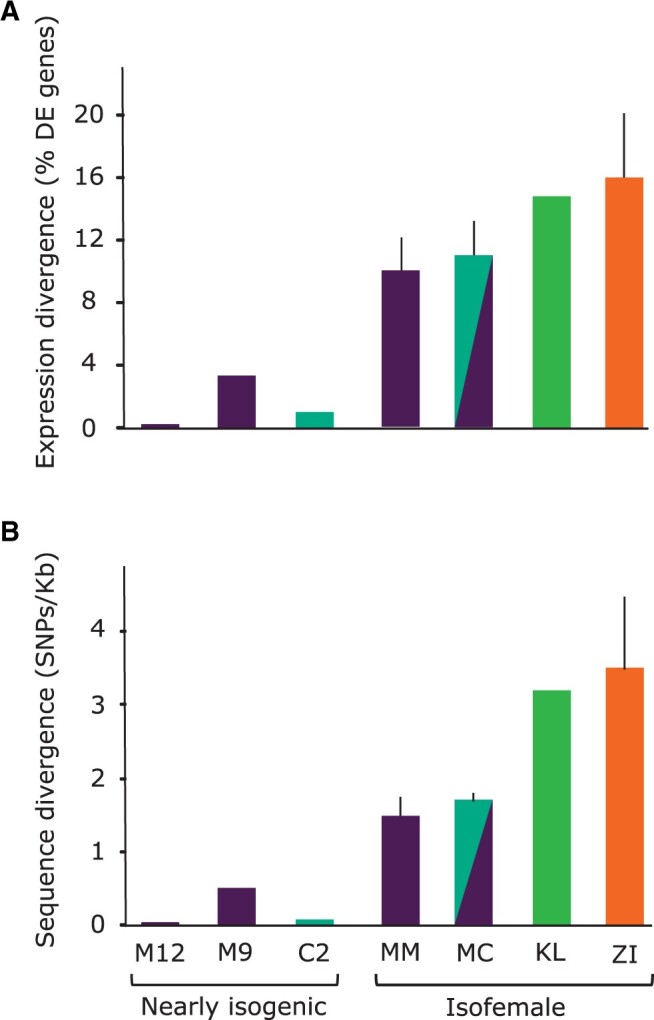
Comparison of variation between nearly isogenic lines from Munich, Germany (M9, M12) and Nicosia, Cyprus (C2), and isofemale lines from Kuala Lumpur, Malaysia (KL), and Siavonga, Zambia (ZI). (A) Gene expression divergence shown as the percentage of differentially expressed (DE) genes at a false discovery rate of 5%. (B) DNA sequence divergence in transcribed regions. “MM” indicates a comparison between the M12 and M9 lines, while “MC” indicates a comparison between both Munich lines and C2. In cases where multiple comparisons were possible, the mean is plotted with error bars indicating the standard deviation.

**Figure 7:**
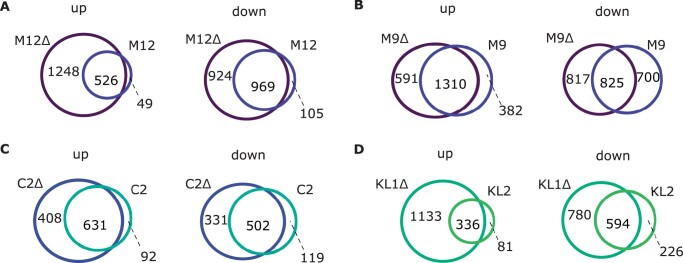
Venn diagram showing the number of shared differentially expressed genes under oxidative stress in deletion (“Δ”) and nondeletion lines in the (A) M12, (B) M9, (C) C2, and (D) KL genetic backgrounds.

Using the full dataset, we detected an overall increase in the expression of metallothionein genes in the oxidative stress samples relative to the controls, with highly significant increases for *MtnA*, *MtnD*, and *MtnE* (Figure  4). To examine if the general increase in *MtnA* could be associated to the indel polymorphism we assessed the expression within each background. We found that the up-regulation of *MtnA* occurred regardless of whether the deletion or nondeletion variant was present in the 3′ UTR (Figure  5), suggesting that *MtnA* plays a general and conserved role in the response to oxidative stress. To better determine the effect of the *MtnA* 3′ UTR indel on expression, we compared the sets of differentially expressed genes within pairs of nearly isogenic lines from Germany (M12 and M9) and Cyprus (C2) that had otherwise homogenized genetic backgrounds, as well as two isofemale lines from Malaysia (KL1 and KL2) that were homozygous for the deletion and nondeletion alleles, respectively. The nearly isogenic lines showed greatly reduced differentiation compared to isofemale lines in both DNA sequence and gene expression (Figure  6), although the extent of this reduction varied among the nearly isogenic pairs. For one of the Munich pairs (M12) genetic differentiation was less than 1% of that between isofemale lines, while for the other pair (M9) it was about 30%. For the Cyprus pair (C2) genetic differentiation was about 4% of that between isofemale lines. In contrast, the isofemale lines from Malaysia (KL1 and KL2), which differ for the *MtnA* indel, also differed greatly throughout their genomes, showing levels of sequence and expression divergence expected for independent, isofemale lines (Figure  6; Supplementary Table S5). As might be expected, there was considerable overlap among the genes responding to stress in the deletion and nondeletion lines in all backgrounds (Figure  7); however, there were significantly more differentially expressed genes (both up- and downregulated) in the deletion lines relative to the corresponding nondeletion lines in all comparisons (Supplementary Table S6). Thus, the *MtnA* 3′ UTR deletion appears to have a consistent effect on the transcriptional response to oxidative stress in a way that is independent of *MtnA* expression (Figure  4) and stress tolerance (Figure  1). A potential explanation for this is that the 3′ UTR deletion may eliminate miRNA binding sites (Catalán *et al.* 2016), freeing up the interacting miRNAs to regulate the expression of other genes. To test this, we examined the expression of target genes regulated by miRNAs predicted to bind within the 49-bp deletion region in the *MtnA* 3′ UTR (Catalán *et al.* 2016). For all of these miRNAs, there was a significant excess of downregulated target genes under oxidative stress (Figure  7), which is consistent with the greater availability of these miRNAs leading to increased negative regulation of their target genes. Furthermore, the downregulated target genes were enriched for those involved in regulatory and transcriptional processes (Supplementary Table S7). Thus, the observed excess of up-regulated genes in the deletion background (Figure  7) could be an indirect effect of miRNA regulation (*i.e.*, the downregulation of miRNA target genes leading to up-regulation of other genes that interact with them).

### Coexpression modules that respond differently to oxidative stress between *MtnA* genotypes

Since we observed that the expression of many genes may be affected by the *MtnA* 3′ UTR indel polymorphism, we modified our WGCNA design to include genotype (deletion or nondeletion) as a factor within treatment. This design revealed modules of coexpressed genes induced by oxidative stress in the deletion background relative to the nondeletion background. Similar to the treatment only design, the major categories were turquoise (autophagy and general stress response) (Supplementary Figure S7A) and blue (metabolism) (Supplementary Figure S7B). However, we observed a decrease in the number of genes associated to each module and an overall reduction in the range of fold-changes, with many genes showing only small expression differences (log_2_ fold-change close to 0) (Supplementary Figure 7, A and B). Our analysis also uncovered two genes that showed an amplification of log_2_ fold-change compared to the treatment only design: *Prosα4T2* (*Proteasome alpha 4 subunit, Testis-specific 2*), which was downregulated (blue module), and *CG31792* (ATPase-coupled transmembrane transporter activity), which was up-regulated (turquoise module), in the deletion background under oxidative stress.

## Discussion

### The effect of the *MtnA* indel polymorphism on oxidative stress tolerance

The *MtnA* 3′ UTR deletion polymorphism previously has been associated with an increase in *MtnA* expression which, in turn, is associated with increased tolerance to oxidative stress (Catalán *et al.* 2016; Ramnarine *et al.* 2019). The previous studies, however, compared isofemale lines that differed not only in their *MtnA* alleles, but also at many loci throughout the genome. This approach makes it impossible to isolate the specific effect of the *MtnA* indel polymorphism on oxidative stress tolerance, or to detect potential interactions with other variants in the genomic background. The latter may be especially relevant, as oxidative stress tolerance is known to be a highly polygenic trait (Weber *et al.* 2012; Guio *et al.* 2014; Harrison *et al.* 2020). In the current study, we measured oxidative stress tolerance using lines from Germany and Cyprus in which the genetic background, aside from the *MtnA* locus, was homogenized through inbreeding. While this homogenization was successful in reducing genetic and expression variation (Figure  6), some residual polymorphism remained, especially in one of the German lines. In addition, we measured oxidative stress tolerance in a pair of inbred lines from Malaysia that differed in their *MtnA* 3′ UTR allele, but did not have a homogenized genomic background, and four independent isofemale lines from Zambia that had the ancestral, nondeletion *MtnA* allele.

Our results indicate that the effect of the *MtnA* deletion on oxidative stress tolerance is highly dependent of the genomic background. In the two pairs of nearly isogenic lines from Germany, the deletion lines showed a significant reduction in mortality under oxidative stress compared to the corresponding nondeletion lines (Figure  1). Although there was a relatively high amount of residual polymorphism between the paired lines in one of the German backgrounds (M9), the concordant results observed in the two backgrounds suggest a consistent effect of the *MtnA* 3′ UTR indel polymorphism on oxidative stress tolerance. However, we cannot rule out a contribution from other polymorphic loci in the genome, especially in the M9 background. In contrast to the German background, the deletion had no detectable effect on oxidative stress tolerance in the Cypriot background, and was associated with a significant increase in mortality under stress in the Malaysian background (Figure  1). Thus, we find evidence for sign epistasis, in which the deletion has opposite effects depending on the genetic background. Note, however, that because the Malaysian background is not homogenized and there is considerable genetic and expression variation between the Malaysian lines (Figure  6), we cannot attribute the observed difference in stress tolerance solely to the *MtnA* indel polymorphism. Interestingly, the benefit of the deletion allele appears to be correlated with its frequency in the tested populations, with the greatest benefit in Germany (frequency* *=* *0.91), no benefit in Cyprus (frequency* *=* *0.65), and a significant cost in Malaysia (frequency* *=* *0.45) (Catalán *et al.* 2016; Ramnarine *et al.* 2019). Thus, it may be that the beneficial effect of the *MtnA* deletion is dependent on an interaction with alleles at other loci that are in high frequency in temperate populations, which could contribute to its clinal distribution (Catalán *et al.* 2016), but confirmation of this trend would require additional experiments with a larger number of populations. The deletion is absent from the ancestral species range in sub-Saharan Africa, aside from rare alleles that appear to be caused by recent admixture with non-African populations (Ramnarine *et al.* 2019), suggesting that it conveys no selective advantage in sub-Saharan populations.

### Transcriptomic data implicate several metabolic processes in oxidative stress response

The genes that show a consistent response to MSB exposure across all populations and genotypes can be considered part of the general oxidative stress response. In the following, we discuss the genes, functional categories, and expression modules that are part of this general response, as revealed by our transcriptomic analyses.

Metallothioneins have been proposed to function as free radical scavengers and as antioxidants against reactive oxygen and nitrogen species (Thornalley and Vasák 1985; Abel and de Ruiter 1989; Cai *et al.* 2000). These genes are typically thought to have distinctive but overlapping functions involving metal ion homeostasis and detoxification (Egli *et al.* 2006; Atanesyan *et al.* 2011; Gaudet *et al.* 2011; Luo *et al.* 2020). In *D. melanogaster*, expression changes in *MtnA* have been demonstrated to be associated with oxidative stress tolerance (Catalán *et al.* 2016; Ramnarine *et al.* 2019). A new finding of the current study is that the expression of *MtnA* and two other metallothionein genes (*MtnD* and *MtnE*) is induced by oxidative stress. Furthermore, *MtnD* and *MtnE* show an even higher induction of expression under stress than *MtnA*, while *MtnB*, *MtnC*, and *MtnF* show no sign of induction (Figure  4). These results suggest that the members of the metallothionein family have diversified in both function and regulation.

GO enrichment analysis revealed a strong connection between oxidative stress and metabolism, specifically redox reactions involving glutathione (GSH), and particularly *glutathione-S-transferase* genes (GSTs). Redox status is often measured as the ratio of GSH (reduced state) to glutathione disulfide (GSSG; oxidized state) and used as a marker for cellular toxicity due to oxidative stress (Bonilla *et al*., 2006). Therefore, GSTs may prove to be of further interest because of their functional association to cellular protection against oxidative stress-inducing agents (Mockett *et al.* 1999; Whitworth *et al.* 2005; Hayes and McLellan 2009). In this context, the mechanism by which MSB affects biological systems may be particularly relevant, as it involves reactive oxygen species generation through quinone redox cycling and/or electrophilic attack involving cellular nucleophiles, both of which are modulated by oxidation or conjugation of glutathione (Akman *et al.* 1985; Baker *et al.* 1996; Wallace *et al.* 2005; Elgawish *et al.* 2013). Treatment with menadione has therefore been associated to oxidative damage due to lipid peroxidation, depletion of cellular glutathione, and variation in expression of glutathione metabolic genes (Chang *et al.* 1992; Kavitha and Chandra 2014; Thomas *et al.* 2016). In addition, changes in the expression of GSTs may be linked to cold tolerance. For example, *Glutathione S-transferase E8* (*GstE8*), which was identified in this study, was also identified as a cold tolerance candidate and as a genotype-environment interaction gene by von Heckel *et al.* (2016). In fact, many of the differentially expressed genes identified by von Heckel *et al.* (2016) and by our study overlap, which provides further evidence for there being shared pathways between cold tolerance and oxidative stress tolerance. It has been previously proposed that at the metabolic level, cold tolerance adaptations may involve changes in neighboring pathways such as those related to redox balance (MacMillan *et al.* 2016). In previous studies, *MtnA* expression showed a variable response to cold stress (MacMillan *et al.* 2016; von Heckel *et al.* 2016; Königer and Grath 2018; Teets and Hahn 2018), which contrasts with the uniform induction of *MtnA* under oxidative stress. This suggests that at least some of the metallothioneins respond specifically to oxidative stress and are not induced by stress in general. Nonetheless, the up-regulated GO term “response to stress” supports the idea that oxidative stress is linked to other stress responses. Within this category, we examined the top 20 differentially expressed genes with the largest log_2_ fold-change. Of these, 10 belong to the HSP gene family. The other genes include: *Ets21C*, *Fst*, *Gadd45*, *mthl7*, *MtnE*, *TotB*, *Cht9*, *GstE1*, *Arc1*, and *CG31659*. With the exception of *GstE1*, these genes are not known for their association to oxidative stress specifically but are involved in varied pathway responses to diverse stress stimuli. Notably, among these genes is one of the few genes implicated specifically in cold tolerance, *Fst* (*Frost*) (Colinet *et al.* 2010), which further suggests a mechanistic relationship between cold stress and oxidative stress.

Among the GO terms with the lowest *P-*values and the greatest downregulation under oxidative stress are a small number of genes related to (1) regulatory processes: *Thymidylate synthase* (*Ts*) and *cardinal* (*cd*); (2) oxidation-reduction and catabolic processes: *beta-hydroxy acid dehydrogenase* (*Had1*), *Stearoyl-CoA 9-desaturase* (*CG9747*), *Alcohol dehydrogenase* (*Adh*), and *Hexokinase-C* (*Hex-C*); and (3) tracheal system development: *Rebuf* (*reb*), *filzig* (*flz*), and *breathless* (*btl*). Notably, previous work has indicated that several genes involved in tracheal system and/or cuticular development were up-regulated with cold acclimation (MacMillan *et al.* 2016). Thus, our observation that genes associated with tracheal system development are downregulated under oxidative stress may imply that the physiological response mechanisms differ between these two stresses. In addition, we observed an up-regulation of genes that were associated to the GO term “proteolysis.” Since several of the top differentially expressed genes in this category are functionally associated to cleavage of peptide bonds and have endopeptidase (*e.g.*, *Jon25Bii*) or protease (*e.g.*, *snk*) activity (Gaudet *et al.* 2011), we hypothesize that the up-regulation of these genes is in response to damage or modification of cellular proteins (Finkel and Holbrook 2000; Das *et al.* 2001; Pickering *et al.* 2013).

A potential limitation of performing GO enrichment analysis with lists of significantly differentially expressed genes is that one primarily observes how an individual gene relates to a functional category. This may overlook correlated expression patterns among genes or genes that have a significant impact based on associations with other genes. Therefore, we re-analyzed our data using WGCNA to identify further candidate genes (Langfelder and Horvath 2008). This approach can assist in the identification of clusters of correlated genes or putative pathways, represented as modules that may be involved in oxidative stress tolerance based on gene expression profiles. Using WGCNA, we can isolate key nodes that have multiple edges, because the malfunction of such genes would affect all connected genes and therefore represent possible interesting candidates for further investigation. In both WGCNA designs (“∼treatment” and “∼genotype: treatment”), the two main modules can be categorized as (1) autophagy and apoptosis (turquoise) and (2) metabolic processes (blue) (Figure  3). In the turquoise module, we observed the up-regulation of genes involved in the general stress response, including a group of highly connected HSP genes and genes related to ubiquitin protein ligase binding (*Ubi-p5E*, *Ubi-p63E*, and *CG11700*), which also connect multiple sub-modules. This may indicate a connection between oxidative stress response genes and those involved in the ubiquitin proteasome pathway (Gaudet *et al.* 2011; Shang and Taylor 2011). On the other hand, in the blue module we observed the gene *glutamate oxaloacetic transaminase* (*Got1*), which is involved in glutamate biosynthesis (Gaudet *et al.* 2011), and *aralar1*, which is a mitochondrial and calcium-binding carrier that catalyzes the calcium-dependent exchange of cytoplasmic glutamate with mitochondrial aspartate across the mitochondrial inner membrane (Gaudet *et al.* 2011). Thus, altered expression of *Got1* and *aralar1* may reflect either a direct effect of the ROS burden and/or a systematic attempt to attenuate oxidative stress conditions through metabolic management of glutamate (Schinder *et al.* 1996; Rival *et al.* 2004; Peng *et al.* 2019). When the *MtnA* genotype is included as a factor, *Prosα4T2* (blue module) and *CG31792* (turquoise module) were revealed as potentially relevant to oxidative stress tolerance in the deletion background relative to the nondeletion background. *Prosα4T2*, involved in proteasomal ubiquitin-independent protein catabolic process, has also been suggested to have important functional roles in spermatogenesis, which may indicate a trade-off between oxidative stress tolerance and male reproduction (Belote *et al.* 1998; Tsakiri *et al.* 2013).

### The effect of the *MtnA* indel polymorphism on gene expression

**Figure 8:**
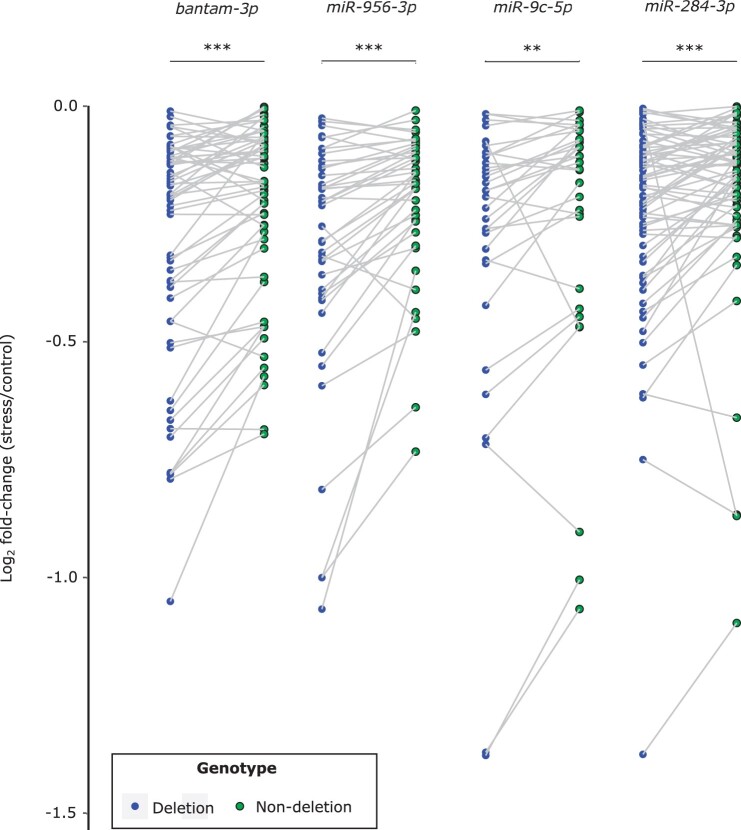
Log_2_ fold-changes (stress/control) of downregulated target genes of miRNAs with predicted binding sites within the *MtnA* 3′ UTR deletion region (*miR-284-3p*, *miR-956-3p*, *miR-9c-5p*, and *bantam-3p*). Two target genes that fall below the limits of the *y*-axis (log2 fold-change <−1.5) are not shown in the plot, but were included in the statistical analysis. Differences between the deletion and nondeletion background were tested using a paired sample Wilcoxon test. **P < 0.01, ***P < 0.001.

In pairwise comparisons of cosmopolitan lines that differed in their *MtnA* indel genotype, we observed that a large portion of the differentially expressed genes were shared between lines, but that there was a greater number of differentially expressed genes under stress in the deletion background than in the nondeletion background (Figure  7). This differential expression did not always correspond to the expression level of the *MtnA* gene (Figure  5), suggesting that the deletion influences the expression of other genes through a mechanism that is independent of its effect on *MtnA*. One possibility is that the polymorphic region of the *MtnA* 3′ UTR contains binding sites for miRNAs, and when this region is deleted the miRNAs are free to regulate the expression of other genes. Consistent with this interpretation, several miRNAs have been predicted to have target sites that overlap with the 49-bp deletion region of the *MtnA* 3′ UTR (Catalán *et al.* 2016). Furthermore, the predicted target genes of these miRNAs show greater downregulation under stress in the deletion background compared to the nondeletion background (Figure  8). This would be expected if there is direct regulation of the target genes by these miRNAs, as miRNAs typically function in the post-transcriptional regulation of gene expression by inducing mRNA degradation (Chen and Rajewsky 2007). However, there was also a significant excess of up-regulated genes under stress in the deletion background. This up-regulation is not likely to be mediated directly by miRNAs, which are predominantly involved in the negative regulation of gene expression. Instead, the up-regulation may be an indirect effect of miRNA regulation. That is, the downregulation of one gene by a miRNA leads to the up-regulation of a second gene through the action of one or more intermediate genes involved in a regulatory network. This interpretation is supported by the finding that genes in the functional categories: “regulation of gene expression” (59 genes), and particularly “negative regulation of gene expression” (35 genes), were overrepresented among the genes that were differentially expressed between deletion and nondeletion lines under oxidative stress (Supplementary Table S7). In addition, many of these genes fall into the general categories with the lowest *P*-values, “regulation of biological processes” (124 genes) and “regulation of cellular processes” (118 genes). One of the more common mechanisms associated to these terms is regulation of transcriptional activity via RNA Polymerase II (Supplementary Table S7), which is a key component in regulating gene expression. Interestingly, there were just three miRNA target genes associated to the ontology term “response to oxidative stress” but with potentially significant impact due to their diverse functionality and effect on transcriptional activity. One of these target genes is *Adar* (*Adenosine deaminase acting on RNA*) an RNA-editing enzyme that has been proposed to facilitate temperature-dependent adaptive evolution through an epigenetic mechanism and downregulation of this gene has been shown to be associated to an increase in expression of genes encoding known ROS scavengers (Chen *et al.* 2004; Gaudet *et al.* 2011; Rieder *et al.* 2015; Duan *et al.* 2017). The other two targets are: *alph* (*alphabet*) a Serine/Threonine specific protein phosphatase that is involved in the mitogen-activated protein kinase (MAPK) cascade and *per* (*period*), a gene essential for biological clock functions (Gaudet *et al.* 2011). *Alph* may represent a potential candidate to investigate our hypothesis concerning the adaptive benefits and indirect regulatory capabilities of the *MtnA* indel-associated miRNAs. *Alph* is involved in the negative regulation of Jun N-terminal kinase (JNK) cascade, therefore downregulation of this gene augments JNK signaling, which induces protective genes that confer increased oxidative stress tolerance (Wang *et al.* 2003; Baril *et al.* 2009). Another example here is the downregulation of *per* which is phenotypically associated to pathological effects on circadian rhythms and aging but may also have metabolic benefits which control longevity (Krishnan *et al.* 2009; Ulgherait *et al.* 2020).

In summary, we have demonstrated that the molecular and genetic mechanisms involved in oxidative stress tolerance are strongly associated to metabolism and DNA/protein damage and repair. Moreover, our transcriptomic data suggest that this response overlaps with genes responding to cold tolerance and that metallothionein genes (*MtnA*, *MtnD*, and *MtnE*) may operate collaboratively in response to oxidative stress. To further investigate the functional elements associated to these two stress responses and their roles in clinal or local adaptation, future studies may utilize combined or parallel oxidative stress and cold stress treatments on lines with precisely controlled genomic backgrounds. In addition, a candidate gene approach involving oxidative and cold stress with single gene knockout/knockdown lines could be employed. With regards to the *MtnA* indel polymorphism and its effect on oxidative stress tolerance, our data imply that the associated phenotype is dependent on alleles at other loci in the genetic background. Therefore, a logical progression of this study may involve experimental work including expression, tolerance, and transcriptomic analyses of a larger number of natural population backgrounds that have been subjected to direct genetic manipulation of the *MtnA* indel region using gene editing techniques. Finally, we provide preliminary evidence for the involvement of miRNAs in the response to oxidative stress, which may be part of the mechanism by which the *MtnA* 3′ UTR deletion affects the expression of *MtnA* and other genes. In the future, reporter genes, small-RNA sequencing, and/or transgenic lines (*e.g.*, UAS-miRNA) could be used to confirm specific miRNA-mRNA interactions and to functionally evaluate the regulatory capabilities of miRNAs associated with the *MtnA* 3′ UTR.

## Data availability

Gene expression data are available at GEO under the accession number GSE174813. All other data are provided in the online supplementary materials are available at figshare: https://doi.org/10.25387/g3.14778540.
